# Supplementary material for: Direct measurement of the 3He+ magnetic moments
Source: Nature. 2022 Jun 8;606(7916):878–83. doi: 10.1038/s41586-022-04761-7 (PMC9242863; doi:10.1038/s41586-022-04761-7)
Supplement: Supplementary file 1 — Supplementary text, figures and tables on the experimental methods and the calculation of the electronic g-factor, shielding parameter, zero-field hyperfine splitting and Zemach radius. [file 41586_2022_4761_MOESM1_ESM.pdf]

nature portfolio

https://doi.org/10.1038/s41586-022-04761-7

Supplementary information

Direct measurement of the He magnetic moments

In the format provided by the authors and unedited

Supplementary Information

1 Supplement - experiment

1.1 Magnetic inhomogeneity

The quadratic inhomogeneity of the magnetic field in the PT $B_{2,PT}$ was determined by exciting the modified cyclotron mode with a resonant signal applied to a split trap electrode and measuring the shift of the axial frequency

$$\frac{\delta\nu_z}{\nu_z} = \frac{1}{m(2\pi\nu_z)^2}\frac{B_2}{B_0}E_+, \tag{S1}$$

as well as the shift of the modified cyclotron frequency

$$\frac{\delta\nu_+}{\nu_+} = \left(-\frac{1}{mc^2} - \left(\frac{\nu_z}{\nu_+}\right)^2 \frac{1}{m(2\pi\nu_z)^2}\frac{B_2}{B_0}\right)E_+. \tag{S2}$$

The latter includes besides the term proportional to $B_{2,PT}$ an additional relativistic contribution, so that $B_{2,PT} = 1.17(4)\ \mathrm{T\,m^{-2}}$ can be extracted from the ratio of both shifts (Fig. S1).

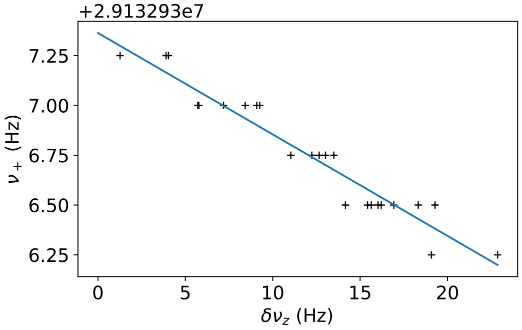

Figure S1: Determination of the magnetic inhomogeneity $B_{2,PT}$. Plotted is the modified cyclotron frequency versus the shift of the axial frequency after excitation of the modified cyclotron mode.

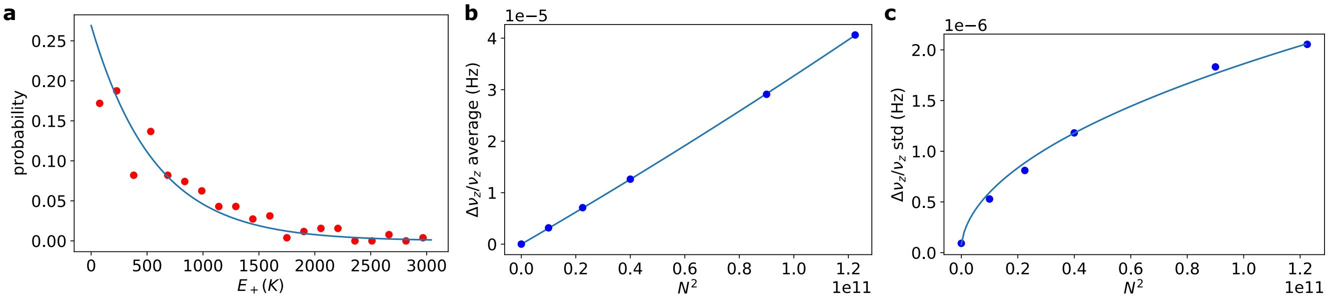

Figure S2: Axial and cyclotron temperature determination. (a): Boltzmann-distribution of the cyclotron energy. (b) and (c): Average and standard deviation of the axial frequency shift after cyclotron excitation in the PT as function of the square of the number N of the excitation cycles.

1.2 Electrostatic anharmonicity

Optimizing the tuning ratio $\mathrm{TR} = \frac{V_c}{V_r}$, defined as the ratio of the voltages applied to the center electrode V_r and the neighbouring correction electrodes V_c, allows to eliminate anharmonic contributions C_4 and C_6 to the electrostatic potential $\Phi(z) = \sum_k C_{2k} z^{2k}$, which would cause energy dependent shifts of the eigenfrequencies [1]. For this purpose the magnetron mode is excited to different energies E_- for different tuning ratios and the resulting shift of the axial frequency

$$\frac{\delta\nu_z}{\nu_z} = \left(\frac{3}{qV_0} \frac{C_4}{C_2^2} - \frac{1}{m(2\pi\nu_z)^2} \frac{B_2}{B_0} \right) E_- \tag{S3}$$

is measured. By this method the anharmonic contributions were minimized to $C_4 = 0(5) \cdot 10^4\ \mathrm{m}^{-4}$ and $C_6 = 2(1) \cdot 10^{-6}\ \mathrm{mm}^{-6}$. The axial frequency shift in Eq. (S3) includes besides the electrostatic anharmonicities also a B_2 dependent term, which, however, is negligible assuming the measured value of $B_{2,PT}$. As a cross-check, the tuning ratio was additionally optimized by heating the axial mode using white noise and measuring the resulting shift of the axial frequency, which is B_2 independent

$$\frac{\delta\nu_z}{\nu_z} = \frac{3}{4} \frac{1}{qV_0} \frac{C_4}{C_2^2} E_z. \tag{S4}$$

The optimal tuning ratios found by both methods agree within 1σ.

1.3 Axial temperature

In order to determine the axial temperature T_z in the PT, the axial and cyclotron modes were coupled in the PT at the sideband. As a result, the cyclotron mode thermalizes with the axial detection system at $T_+ = T_z \frac{\nu_+}{\nu_z}$. The ion is afterwards transported to the AT where the axial frequency is measured. Due to the magnetic bottle in the AT, the axial frequency is shifted proportional to the cyclotron energy $E_+ = k_B T_+$, see Eq. (S1). A maximum likelihood fit of a Boltzmann-distribution to the axial frequencies therefore gives the temperature T_+ and thereby

$T_z = 11.7(7)$ K. As a cross-check, the axial temperature in the PT was additionally determined by exciting the modified cyclotron mode and afterwards measuring the axial frequency in the PT. After repeating this 200 times, the temperature can be calculated from the standard deviation and average of the axial frequency distribution as described in [2]. This method requires inserting $D_4 = dC_4/d\mathrm{TR}$ as obtained theoretically from the trap geometry $D_4 = -3.5 \cdot 10^9$ m^{-4} and the measured $B_{2,PT}$ and gives a consistent result for the axial temperature $T_z = 12(1)$ K, see Fig. S2.

1.4 Time standard

All frequency generators and FFT signal analyzers are locked with a Stanford Research Systems FS725 Rubidium 10 MHz frequency standard. As the determination of E_{HFS} requires an absolute frequency measurement, the Rubidium standard is itself locked to a GPS pps signal.

1.5 Lineshape

Due to the oscillation of the ion in the residual magnetic bottle in the PT $B_{2,PT}$, the resonance frequency varies during the spin excitation time $T_{exc} = 120$ s. Accordingly, the linewidth parameter $\delta\omega(B_{2,PT}, T_z)$ characterizing the spread of the resonance frequency is dependent on the magnetic bottle and the average axial amplitude $\langle z^2 \rangle \propto T_z$. The resultant lineshape is derived by Brown [3] and Verdu [4] to be $\chi_1(\Delta, \gamma, \delta\omega)$ and $\chi_2(\Delta, \Omega, \delta\omega)$, respectively. Here, Ω is the Rabi frequency and γ is the coupling of the ion to the axial resonator. The correspondent spin-flip probability $P_{1/2}(\Delta)$ as a function of the detuning $\Delta = \nu_{RF} - \nu_L$ of the applied drive frequency ν_{RF} from the resonance frequency ν_L is then

$$P_{1/2}(\Delta) = \frac{1}{2}\left(1 - \exp\left(-\frac{1}{2}\Omega T_{\mathrm{exc}} \chi_{1/2}\right)\right). \tag{S5}$$

The expected resonance frequency $\nu_L(B)$ follows from the double-dip measurement of the cyclotron frequency, which is normally distributed with a relative width of $1.7 \cdot 10^{-9}$. The underlying function describing the spin-flip probability is therefore convoluted with the Gaussian of the magnetic field measurement. For small Rabi frequency Ω or small $B_{2,PT}T_z$, the jitter of the magnetic field measurement dominates and the spin-flip probability can be approximated as a Gaussian. In order to estimate the systematic uncertainty due to the unknown deviation from a Gaussian profile, the two possible spin-flip probabilities $P_{1/2}(\Delta)$ convoluted with a Gaussian are fitted with a Gaussian line to extract the shift of the center-frequency and the correspondent difference of the deduced g factors and E_{HFS} (Fig. S5).

The fitted Rabi frequencies are different for both lineshapes and shown for the electronic transitions $|1\rangle \leftrightarrow |3\rangle$ and $|2\rangle \leftrightarrow |4\rangle$ in Fig. S6 and for the nuclear transitions $|1\rangle \leftrightarrow |2\rangle$ and $|3\rangle \leftrightarrow |4\rangle$ in Tab. S1. Each measurement is ascribed a systematic uncertainty given by the maximum of the differences between a Gaussian fit and a fit with

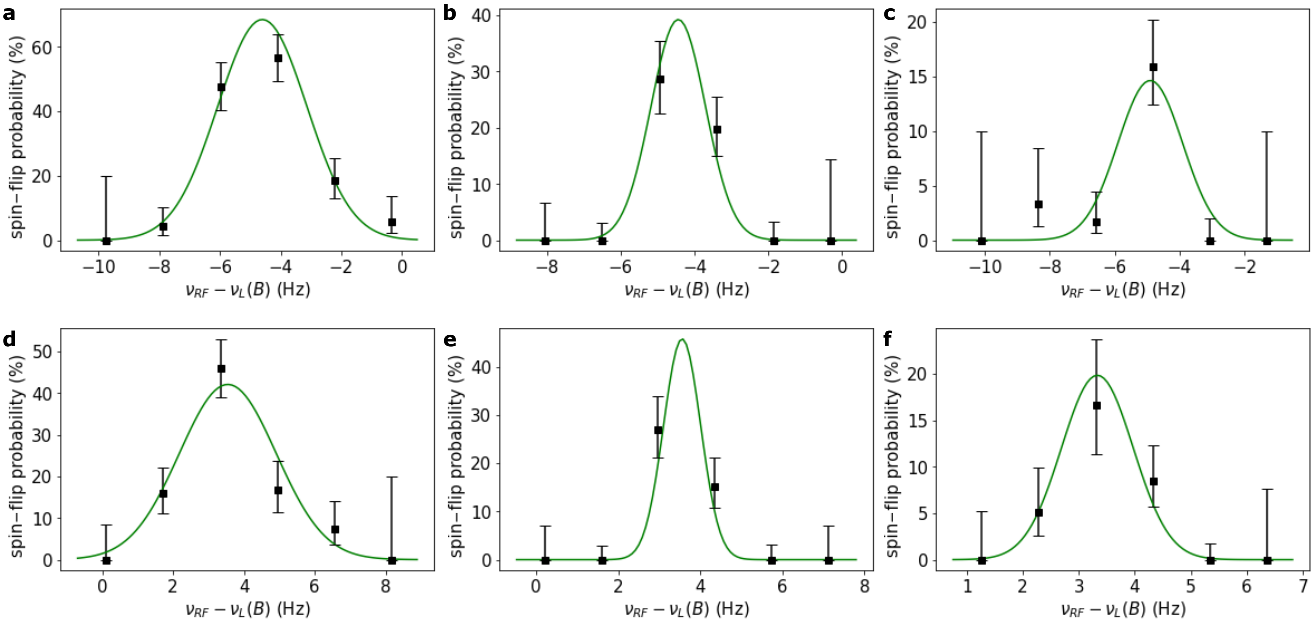

Figure S3: Nuclear transitions at different microwave powers. (a-c): transition $|1\rangle \leftrightarrow |2\rangle$ at -20dbm, -33dbm and -40dbm. (d-f): transition $|3\rangle \leftrightarrow |4\rangle$ at -15dbm, -23dbm and -30dbm.

the functions $P_{1/2}(\Delta)$ convoluted with a Gaussian. The final result for g_e is calculated as the weighted average of the measurements at the three smallest Rabi frequencies, i.e. smallest systematic uncertainties. These include two resonances of transition $|2\rangle \leftrightarrow |4\rangle$ and one of transition $|1\rangle \leftrightarrow |3\rangle$. Similarly, the final results for g_I and E_{HFS} only take into account the measurement at the smallest amplitude, see Fig. S3 and Fig. S4.
Thus, the results for the electronic and nuclear parameters include 1540 and 490 data points, respectively, with two data points measured per hour in both cases.

The coupling of the axial and modified cyclotron modes during the double-dip measurement leads to a modulation of the axial oscillation amplitude and thus of $\delta\omega$ with the Rabi frequency of the coupling $\Omega_{DD} = 22$ Hz, causing sidebands in the resonance lineshape. A significant shift due to this effect was excluded by measuring the $|2\rangle \leftrightarrow |4\rangle$ resonance once with and without a double-dip during the spin-flip drive at the same microwave power, giving results that agree within 1σ. For this purpose the cyclotron frequency measurement during the spin-flip drive was replaced by the average of the measured frequencies before and after applying the drive, as these values were consistent for all measurement runs.

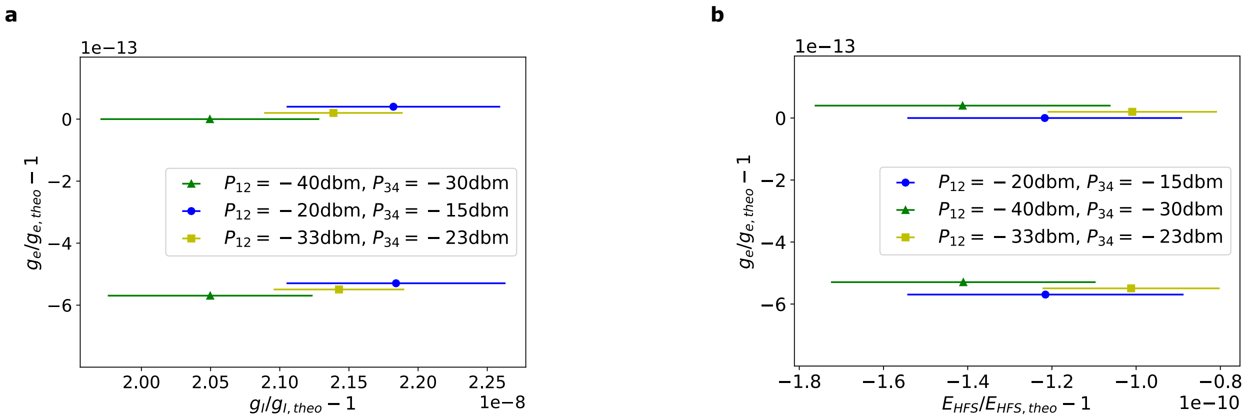

Figure S4: Results for the shielded nuclear g factor and hyperfine splitting with a maximum likelihood fit of a Gaussian, combining one resonance of each nuclear transition with microwave powers P_{12} and P_{34}. As the electron g-factor is known precisely by theory, for the final result g_e is fixed to the theoretical value in the fits of the nuclear resonances. Plotted are the results if $g_e = g_{e,theo}$ is assumed and when g_e is changed by 3σ of the theoretical value, i.e. $g_e/g_{e,theo} - 1 = -6 \cdot 10^{-13}$ for all microwave powers. In order to make the overlapping errorbars better visible, the datapoints are depicted slightly shifted along the y-axis. Changing g_e by 3σ shifts the fitted g_I' and $E_{\rm HFS}$ by two orders of magnitude less than their statistical uncertainties.

Table S1: Fit results for the Rabi frequencies Ω_1 and Ω_2 of the two measured nuclear transitions assuming lineshapes χ_1 and χ_2, respectively, which correspond to a systematic uncertainty shown in Fig. S5. As g_I and $E_{\rm HFS}$ are determined from a combined fit of one resonance of each nuclear transition, the combined uncertainty of both transitions are given for these parameters.

amplitude (dbm)	transition	Ω_1 (1/s)	Ω_2 (1/s)	$\delta g_I/g_I$	$\delta E_{\rm HFS}/E_{\rm HFS}$
−20	12	3.8(7)	0.76(7)		
−15	34	1.9(4)	0.53(6)	$7 \cdot 10^{-10}$	$5 \cdot 10^{-12}$
−33	12	0.9(2)	0.29(5)		
−23	34	0.5(1)	0.20(4)	$5 \cdot 10^{-10}$	$1 \cdot 10^{-12}$
−40	12	0.33(8)	0.15(3)		
−30	34	0.30(8)	0.13(3)	$4 \cdot 10^{-10}$	$1 \cdot 10^{-12}$

2 Supplement - theory

2.1 Theoretical contributions to the electron g_e factor

The g_e factor for ^{3}He$^+$ was calculated similarly to that of ^{4}He$^+$ in Ref. [5]. The contributing terms are shown in Table S2. The most notable difference between the two isotopes is the recoil contribution due to a different nuclear mass. Calculating the nuclear mass using the GRASP code [6], with the He-3 mass from the recent atomic mass evaluation [7] as input parameter, resulted in recoil corrections which are identical to all digits specified to results obtained with the helion mass from [8]. The result for the leading recoil correction to $\mathcal{O}\left((Z\alpha)^2\right)$ [9–11] differs from the all-order in $Z\alpha$ result [12] given in Table S2 by 1.18×10^{-12}. For the calculation of the higher-order in $\frac{m}{M}$ recoil corrections, we used a formula which is all-order in the mass ratio [10] (later confirmed in [13, 14]). We note

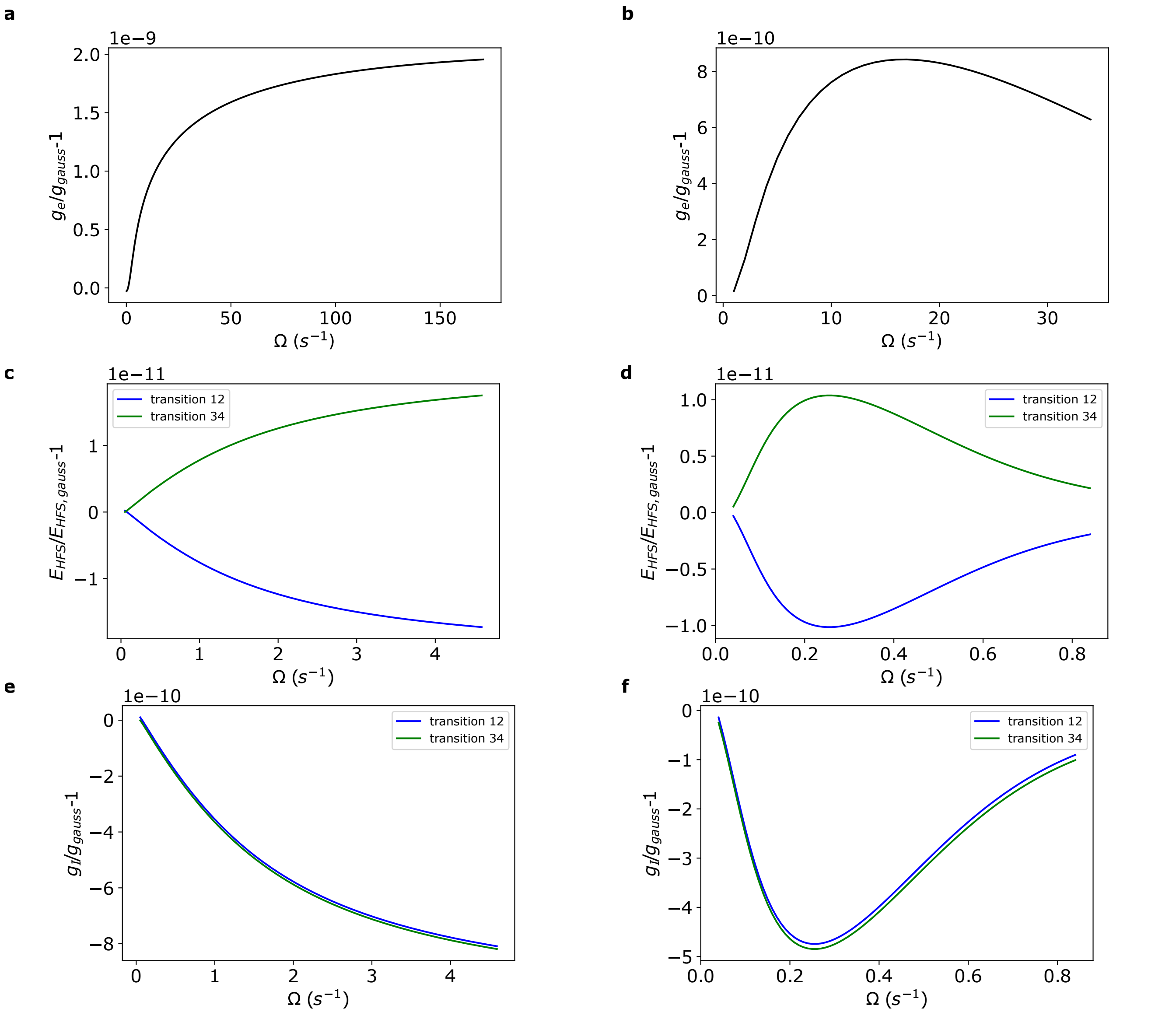

Figure S5: Difference of the fitted electron g-factor, nuclear g-factor and E_{HFS} with the models P_1 (A,C,E) and P_2 (B,D,F) compared to a Gaussian as a function of the Rabi frequency extracted from the fit.

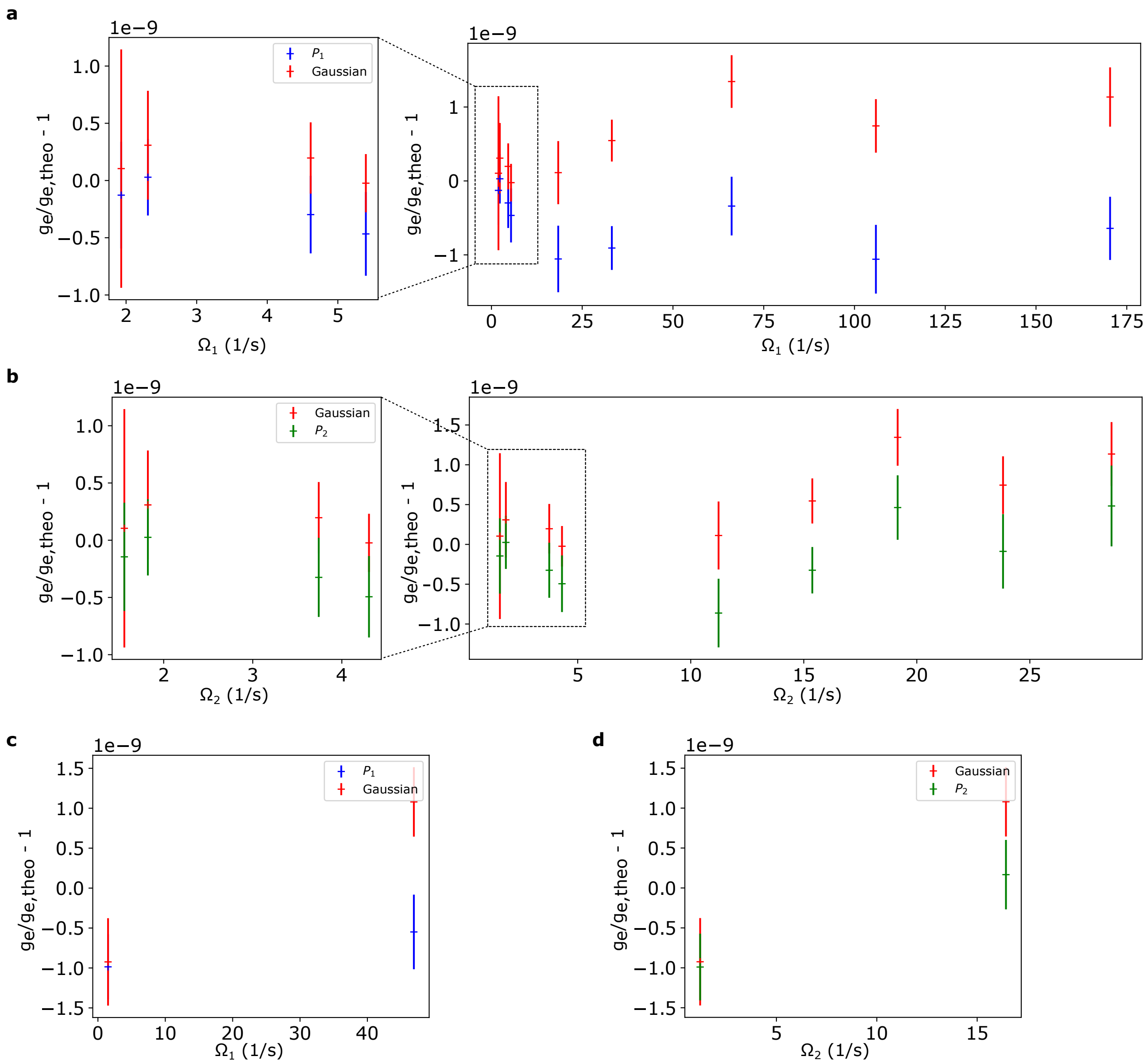

Figure S6: Fit results with different lineshape models. (a) and (b): transition $|2\rangle \leftrightarrow |4\rangle$. (c) and (d): transition $|1\rangle \leftrightarrow |3\rangle$. Shown is the electron g-factor fitted with a Gaussian or functions $P_{1/2}$ from Eq. (S5) convoluted with a Gaussian. The x-axis is the Rabi frequency fitted with the respective lineshapes. The graphs on the left side in (a) and (b) show only the smallest Rabi frequencies, where the results for different lineshapes agree.

here that the result up to $\mathcal{O}\left(\left(\frac{m}{M}\right)^2\right)$ [9, 11] deviates by only 1×10^{-14} from the all-order result. Apart from that, several recent results were taken into account. Free-QED contributions have been reevaluated, namely, the four-loop coefficient was evaluated to high precision in Ref. [15], and the five-loop term has been updated in Ref. [16]. Recently computed two-loop binding contributions of $\mathcal{O}\left((Z\alpha)^5\right)$ [17, 18] are very small for $Z = 2$. Higher-order corrections to two-loop magnetic loop vacuum polarization diagrams [19] turned out to be negligibly small for $Z = 2$. Binding corrections of order $(Z\alpha)^2$ were calculated using a simple formula from Ref. [20], which is a confirmation of earlier derivations of this quantity [9–11]. The uncertainty due to uncalculated binding corrections of order $(Z\alpha)^4$ and higher to Feynman diagrams with at least three loops was estimated by adapting a method from Ref. [21]. The uncertainty due to uncalculated binding corrections of order $(Z\alpha)^6$ and higher to Feynman diagrams with at two loops was calculated using the methods from [21] and [17], with the larger of the two estimates given as the uncertainty of this contribution in Table 2. The FS correction was calculated nuclear model independently using analytic formulas from [22–24] and the rms charge radius from [25]. The muonic vacuum polarization correction [26] as well as our estimates for the nuclear deformation [27], nuclear susceptibility [28] and our calculated nuclear polarization value are also much smaller than 1 in the last digit shown in Table S2. Finally, the latest all-order evaluation of the one-loop self-energy contribution has been included [29]. With this, the uncertainty of the total g_e factor is dominated by the uncertainty of α.

It is worth pointing out that an independent calculation of the 5-loop contribution to the free electron anomaly [30] is in disagreement with the result from [8] by four times its uncertainty as specified in [8].

Our calculation of the bound-electron g factor relies on the latest CODATA value for the fine-structure constant. It is worth mentioning that since the CODATA 2018 compilation, α has been measured with a better accuracy than specified in CODATA 2018 [31]. This new value of α disagrees with the CODATA value by 5 σ. However, since the uncertainty due to the (CODATA) fine structure constant is three orders of magnitude smaller than the experimental uncertainty of the work presented here, we do not expect this deviation to be relevant for this work.

2.2 Theory of the hyperfine splitting

The energy difference between the hyperfine levels of the ground state, with the total angular momenta $F = 0$ and $F = 1$ (electronic and nuclear spins oriented anti-parallel and parallel, respectively), is

$$E_{\mathrm{HFS}} \equiv \Delta E_{\mathrm{HFS}}(F=1) - \Delta E_{\mathrm{HFS}}(F=0)\,, \tag{S6}$$

Contribution		$^3He^+$	Following Refs.
Dirac value		-1.999 857 988 825 25(4)	[32]
Finite size		-0.000 000 000 003 16(5)	TW, [22–25]
1-loop QED	$(Z\alpha)^0$	-0.002 322 819 465 77(35)	[8, 33]
	$(Z\alpha)^2$	-0.000 000 082 462 19	[9–11, 20]
	$(Z\alpha)^4$	-0.000 000 001 976 70	[21, 34]
	$(Z\alpha)^{5+}$ SE	-0.000 000 000 035 15(5)	[29]
	$(Z\alpha)^{5+}$ VP-EL, Uehling	-0.000 000 000 002 52	[35–37]
	$(Z\alpha)^{6+}$ VP-EL, WK (incl. FS)	-0.000 000 000 000 01(1)	[37]
	$(Z\alpha)^{5+}$ VP-ML	-0.000 000 000 000 16	[37, 38]
	remaining 1-loop-FS	0.000 000 000 000 00(1)	[22, 25]
2-loop QED	$(Z\alpha)^0$	0.000 003 544 604 50	[8, 39, 40]
	$(Z\alpha)^2$	0.000 000 000 125 84	[9–11, 20]
	$(Z\alpha)^4$	-0.000 000 000 002 41	[21]
	$(Z\alpha)^4$, LBL	0.000 000 000 000 39	[41]
	$(Z\alpha)^5$ SESE	-0.000 000 000 000 02	[17]
	$(Z\alpha)^5$ LBL	-0.000 000 000 000 01	[17, 18]
	$(Z\alpha)^5$ ML	-0.000 000 000 000 00	[17]
	$(Z\alpha)^{5+}$ S(VP)E, SEVP, VPVP	-0.000 000 000 000 00(10)	[42], interpolation
	$(Z\alpha)^{6+}$ SESE	-0.000 000 000 000 00(3)	[17, 21]
$\geq$ 3-loop QED	$(Z\alpha)^0$	-0.000 000 029 497 84(3)	[8, 15, 16, 43–45]
	binding	-0.000 000 000 001 05(5)	[9–11, 20, 21]
Recoil	$\frac{m}{M}$, all-order $Z\alpha$	-0.000 000 038 758 43	[6, 7, 9–12]
	h.o $\frac{m}{M}$	0.000 000 000 021 15	[9–11, 13, 14]
	rad-rec	0.000 000 000 030 01(6)	[9–11, 14]
Hadronic	$(Z\alpha)^0$	-0.000 000 000 003 39(3)	[8, 46–48]
	$(Z\alpha)^2$	0.000 000 000 000 00	[9–11, 20]
Electroweak		-0.000 000 000 000 06	[8]
Sum		-2.002 177 416 252 23(39)	

Table S2: The bound-electron g_e factor in $^3He^+$: contributions to g_e. TW stands for results calculated in this work.

with (see Ref. [37, 49])

$$\Delta E_{\text{HFS}}(F) = \alpha g_I \frac{m_e}{m_p} \frac{F(F+1) - I(I+1) - j(j+1)}{2j(j+1)} m_e c^2 (Z\alpha)^3 \mathcal{M} \times \left[A(Z\alpha) + \delta_{\text{FS}} + \delta_{\text{NP}} + \delta_{\text{QED}} + \delta_{\mu\text{VP}} + \delta_{\text{hadVP}} + \delta_{\text{ew}} + \delta_{\text{recoil}}\right] , \tag{S7}$$

with the quantities defined in the manuscript. This leads to

$$E_{\text{HFS}} = E_F \left[A(Z\alpha) + \delta_{\text{FS}} + \delta_{\text{NP}} + \delta_{\text{QED}} + \delta_{\mu\text{VP}} + \delta_{\text{hadVP}} + \delta_{\text{ew}} + \delta_{\text{recoil}}\right] , \tag{S8}$$

with

$$E_F = \alpha g_I \frac{m_e}{m_p} \frac{4}{3} m_e c^2 (Z\alpha)^3 \mathcal{M} \, . \tag{S9}$$

Here, $I = \frac{1}{2}$ is the nuclear spin [50], and the angular momentum of the electron is $j = \frac{1}{2}$. The relativistic factor for the 1s state is

$$A(Z\alpha) = \frac{2\gamma + 1}{\gamma(4\gamma^2 - 1)} , \tag{S10}$$

with $\gamma = \sqrt{1 - (Z\alpha)^2}$, and the mass factor is $\mathcal{M} = \left(1 + \frac{m_e}{M_N}\right)^{-3}$, with M_N being the nuclear mass.

An approach to express the finite-size correction δ_{FS} to the HFS for low Z was developed in Refs. [51, 52], $\delta_{\text{FS}} = -\delta_{\text{E}} - \delta_{\text{M}}$, with δ_{E} and δ_{M} corresponding to the finite size charge and magnetization distribution corrections, respectively. The formula for the combined effect, as derived in Refs. [51, 52], is as follows:

$$\delta_{\text{E}} + \delta_{\text{M}} = (\delta_{\text{E}}^{\text{nr}} + \delta_{\text{M}}^{\text{nr}})(1 + \delta_{\text{rel1}}) + \delta_{\text{rel2}} \, . \tag{S11}$$

The sum of the leading nonrelativistic corrections $-\delta_{\text{E}}^{\text{nr}} - \delta_{\text{M}}^{\text{nr}}$ is then identified as the Zemach correction [53]

$$\delta_{\text{Zemach}} = -2\alpha Z \frac{m_e M_N}{m_e + M_N} r_{\text{Z}} \, . \tag{S12}$$

The Zemach radius is defined as

$$r_Z = -\frac{4}{\pi} \int_0^\infty \frac{\mathrm{d}q}{q^2} \left(\frac{1}{1+\kappa} G_E(q^2) G_M(q^2) - 1 \right) , \tag{S13}$$

with the electric $G_E(q^2)$ and magnetic $G_M(q^2)$ form factors of the nucleus. $G_M(0) = 1 + \kappa$, with the anomalous magnetic moment of the nucleus defined as [54]

$$\frac{1+\kappa}{M_N} = \frac{g_I}{2Zm_p} \, . \tag{S14}$$

The dominant part of the total FS correction can therefore be calculated nuclear model independently using the Zemach radius from the literature [25]. The leading Zemach correction is $\delta_{\text{Zemach}} = -0.0001911(12)$. For the relativistic corrections, the following formulas were derived in Refs. [51, 52]:

$$\delta_{\text{rel1}} =(Z\alpha)^2 \left(\frac{7}{4} - \gamma - \ln(2Z\alpha)\right) , \tag{S15}$$

$$\begin{aligned}\delta_{\text{rel2}} =& -2(Z\alpha)^3 m_e \langle r\rangle_E \left(\frac{\langle r\ln(m_e r)\rangle_E}{\langle r\rangle_E} - \frac{839}{750}\right) - \\ &\frac{(Z\alpha)^3 m_e R_0}{5}\left(\frac{3\langle r^4\rangle_M}{2R_0^4} - \frac{19\langle r^6\rangle_M}{42R_0^6} + \frac{19\langle r^8\rangle_M}{360R_0^8} - \frac{2\langle r^{10}\rangle_M}{825R_0^{10}}\right) - \\ &(Z\alpha)^3 m_e R_0 \left(\frac{\langle r^2\rangle_M}{R_0^2} - \frac{\langle r^4\rangle_M}{10R_0^4}\right)\left(\ln(m_e R_0) + \frac{1}{30}\right) .\end{aligned} \tag{S16}$$

Here, γ is the Euler constant, and $R_0 = \sqrt{\frac{5}{3}\langle r^2\rangle_E}$. Results obtained with these formulas for a homogeneously charged sphere model for low-Z nuclei are in good agreement with tabulated results in Ref. [49]. In addition to the relativistic effects, a formula for radiative corrections to the nuclear finite size δ_{rad} based on the exponential charge and magnetization distribution model was derived in Ref. [55]. It is

$$\delta_{\text{rad}} = -\delta_{\text{Zemach}}\frac{\alpha}{3\pi}\left(4\ln(m_e R_D) + \frac{4111}{420}\right) , \tag{S17}$$

with $R_D^2 = \frac{\langle r^2\rangle_E}{12}$.

For the calculation of relativistic corrections as derived in Ref. [51], we relied on different models of the charge and magnetization distributions. In Ref. [56], parametrizations of both charge and magnetization distributions are given which are based on elastic scattering data. In Ref. [57], a charge distribution is parametrized as a sum of Bessel functions. To obtain an estimate for the nuclear model uncertainty, we also calculated the relativistic corrections using different charge and magnetization model distributions suggested for light elements in Ref. [51], like the exponential and Gaussian distributions. While using these distributions, we adjusted the model parameters such that the rms charge $\sqrt{\langle r^2\rangle_E} = 1.973(14)$ fm and magnetic radii $\sqrt{\langle r^2\rangle_M} = 1.976(47)$ fm match the values given in Ref. [25]. In an attempt to give a conservative estimate for the upper boundary of the nuclear model uncertainty, we also used several nuclear models which are more unrealistic for low Z, that is, the homogeneous sphere and the two-parameter Fermi distribution with a skin thickness parameter of 2.3 fm [37]. For all models, relativistic corrections were similar, ranging from -2.82×10^{-6} to -2.87×10^{-6}. We would like to point out that the nuclear-model-dependent part of the relativistic corrections [equation (16)] amounts to less than 10%, namely, -0.22×10^{-6} to -0.27×10^{-6}. This total model-dependent part of the relativistic corrections is also significantly smaller than the uncertainty due to the Zemach radius. We estimated our error due to inconsistent usage of nuclear model parameters from different references as the maximum difference between any two results obtained with the

above mentioned models. This error of 5×10^{-8} is much smaller than the uncertainty due to the Zemach radius. The uncertainty due to the rms and magnetic radii given in Ref. [25] are 5×10^{-9} and 2×10^{-9}, respectively. Furthermore, we estimated the uncertainty due to uncalculated higher orders in $Z\alpha$ to be 1.5×10^{-8} . In total, we find the following finite-size correction to the hyperfine splitting

$$\delta_{\mathrm{FS}} = -0.0001939(12) \,. \tag{S18}$$

Here, the bracket shows the uncertainty due to the Zemach radius, whereas the uncertainty originating from the difference between nuclear models is much smaller. As can be seen from equations (S12) and (S16), nuclear model-dependent quantities, i.e. the above mentioned moments of charge and magnetization distributions, are suppressed by a factor $(Z\alpha)^2$ compared to the leading Zemach term, explaining the small nuclear model uncertainty. The total finite nuclear size correction corresponds to a frequency shift of 1.678 MHz, the uncertainty due to the Zemach radius being 11 kHz.

The QED correction to the hyperfine splitting is [58]

$$\delta_{\mathrm{QED}} = a_e + \delta_{\mathrm{QED,bind}} \,. \tag{S19}$$

Here, a_e is the free-electron magnetic anomaly. Binding corrections $\delta_{\mathrm{QED,bind}}$ at the one-loop level were calculated to all orders in $Z\alpha$ using formulas and tabulated values obtained for point nuclei in Refs. [37, 59]. We assumed an uncertainty of 1 in the last digit given where no uncertainty was specified in these references. At the two-loop level, binding corrections were taken into account up to $\mathcal{O}\left((Z\alpha)^2\right)$ [52, 58, 60]. The uncertainty of the numerical coefficients in these references corresponds to an uncertainty of δ_{QED} of 4×10^{-9}. We estimate the uncertainty of δ_{QED} due to uncalculated higher-order QED corrections to be 6×10^{-9}. Muonic $\delta_{\mu\mathrm{VP}}$ and hadronic δ_{hadVP} vacuum polarization corrections were estimated using formulas from Refs. [55, 61, 62]. The electroweak interaction contribution δ_{ew} was calculated using formulas from Ref. [63].

For the calculation of the recoil correction to order $\mathcal{O}(Z\alpha)$ [52, 55], we use the approach developed for the hydrogen atom in Ref. [64–67]. This formalism depends on the electric and magnetic form factors of the nucleus.We calculated the recoil correction using model form factors from Ref. [56] which are based on elastic electron scattering experiments. We also used model formulas for form factors previously used in hyperfine splitting calculations for the hydrogen atom [52, 68]. In this case, we adjusted the model formulas such that the rms $\langle r \rangle_E^2 = -6 \, \frac{\mathrm{d}}{\mathrm{d}q^2} G_E(q^2)\Big|_{q^2=0}$ and magnetic radii $\langle r \rangle_M^2 = -\frac{6}{1+\kappa} \, \frac{\mathrm{d}}{\mathrm{d}q^2} G_M(q^2)\Big|_{q^2=0}$ correspond to the values given in Ref. [25]. In order to find a conservative estimate for the upper boundary for the nuclear model uncertainty, we also used model form factors which correspond to the Fourier transform of the charge and magnetization distribution of the homogeneous sphere model. The difference between recoil calculations for different model form factors is the first uncertainty given

Contribution	parameter		energy [kHz]		following Refs.
Fermi energy			-8656528		[8, 37]
Relativistic factor	$A(2\alpha)-1$	0.000 319 60	-2767		[37]
Finite size	$\delta_{\rm FS}$	$-0.000\,193\,9(12)$	1678	(11)	TW, [51, 52, 55]
Nuclear polarization	$\delta_{\rm NP}$	0.000 000 01	-0	(11)	TW
QED	$\delta_{\rm QED}$	0.000 946 90(1)	-8197		[8, 37, 52, 58–60]
Muonic VP	$\delta_{\mu{\rm VP}}$	0.000 000 12(12)	-1	(1)	[55, 61]
Hadronic VP	$\delta_{\rm hadVP}$	0.000 000 08(8)	-1	(1)	[55, 61]
electroweak	$\delta_{\rm ew}$	0.000 000 07	-1		[63]
recoil	$\delta_{\rm recoil}$	$-0.000\,013\,19(25)(6)(115)(11)$	114	(2)(1)(10)(1)	TW, [52, 62, 65–67]
Sum			-8665701	(19)	
experiment			-8665649	$.86577(26)_{stat}(1)_{sys}$	TW

Table S3: Contributions to the ground-state hyperfine splitting in $^3{\rm He}^+$, in units of kHz. Uncertainties which are smaller than 1 in the last digit are usually not shown. TW stands for results calculated in this work.

in Table 3. We assume that by using different model form factors, including the unrealistic Fourier transform of the homogeneous sphere model, we overestimate the nuclear model uncertainty rather than underestimating it. (Especially, since the model uncertainty in Table 3 is larger than the uncertainty due to fit parameters from Ref. [56].) We calculated the uncertainties of the recoil parameter $\delta_{\rm recoil}$ due to the uncertainty of the magnetic radius to be 6×10^{-8} (second uncertainty in Table S3). The uncertainty due to the charge radius is too small to be shown in Table S3. The higher-order recoil correction to order $\mathcal{O}\left((Z\alpha)^2\right)$ [52] was calculated using formulas developed for the hydrogen atom in the point-nucleus approximation in Ref. [65] by replacing $\alpha \to Z\alpha$. To account for uncalculated higher orders, we assign a 100% uncertainty to this contribution (third uncertainty in Table S3). We would like to point out that the nuclear model uncertainty is smaller than the uncertainty due to uncalculated higher orders. Calculating the radiative recoil parameter using equation (2) in Ref. [62], we obtain 1.1×10^{-7}, which corresponds to 1 kHz. Since this formula is developed for a structureless nucleus and therefore not applicable for $^3{\rm He}^+$, we use this result as an estimate of the uncertainty due to radiative recoil (fourth uncertainty in Table S3).

Another nuclear structure contribution originates from nuclear polarization. The evaluation has been performed in the framework of a semi-analytical model, introduced in Ref. [69] and later used in Refs. [70–73]. In our recent work [74] we applied this formalism to calculations of NP corrections to the energies and g factor of H-like ions. In the case of HFS, the NP correction can be expressed analogously by the formula:

$$\Delta E_a^{\rm NP,HFS} = -\alpha \sum_{LM}\sum_{n,k} B(EL) \int_{-\infty}^{\infty} \frac{d\omega}{2\pi i} \frac{2\omega_L}{\omega^2-\omega_L^2+i0} \times \left[2\frac{\langle a|F_L Y_{LM}^*|n\rangle\langle n|F_L Y_{LM}|k\rangle\langle k|H_\mu|a\rangle}{(\epsilon_a-\omega-\epsilon_n(1-i0))(\epsilon_a-\epsilon_k(1-i0))} + \frac{\langle a|F_L Y_{LM}^*|n\rangle\langle n|H_\mu|k\rangle\langle k|F_L Y_{LM}|a\rangle}{(\epsilon_a-\omega-\epsilon_n(1-i0))(\epsilon_a-\omega-\epsilon_k(1-i0))} \right]. \tag{S20}$$

Here, the summation goes over all nuclear excitations with energies ω_L and reduced transition probabilities $B(EL)$. The Hamiltonian $H_\mu = \frac{e}{4\pi}\boldsymbol{\mu}\cdot\frac{\boldsymbol{r}\times\boldsymbol{\alpha}}{|\boldsymbol{r}|^3}$ describes the interaction of the electron at the coordinate vector $\boldsymbol{r}$ with the

nuclear magnetic moment $\boldsymbol{\mu}$, with the $\boldsymbol{\alpha}$ being the usual 3-vector of Dirac matrices. n and k denote here the electronic states in the spectrum of the bound Dirac equation, the Y_{LM} are spherical harmonics, and the radial functions F_L are given in the sharp surface approximation [69] as

$$F_0 = \frac{5\sqrt{\pi}}{2R_0^3}\left[1-\left(\frac{r}{R_0}\right)^2\right]\Theta(R_0-r)\,,$$
$$F_L = \frac{4\pi}{(2L+1)R_0^L}\frac{\min(r,R_0)^L}{\max(r,R_0)^{L+1}}\,, \qquad L \geq 1\,, \tag{S21}$$

with R_0 being the nuclear radius. The first term in the brackets corresponds to the irreducible (for $k \neq a$) and reducible ($k = a$) contributions, and the second one to the vertex contribution. The factor of 2 in the first term originates from the existence of two equivalent diagrams. We would like to stress that the used formalism rigorously accounts for the electronic structure and spectrum, which is important in the case of the hyperfine interaction.

The nuclear parameters ω_L and $B(EL)$ for nuclear states have been calculated in the framework of the random phase approximation (RPA) with Skyrme-type nuclear interactions [75, 76]. Recent calculations [77] of the NP correction to the energy levels for different models showed the model dependence on the $\approx 15\%$ level for heavy muonic atoms. The results are sensitive to the nuclear radius and to the use of a smoothing function $F_{\rm BW}$ [78], which allows to remove the singularity of the H_μ potential at the origin. The results for the relative NP corrections to the HFS are presented in Table S4. A comparison with other literature values for nuclear polarization corrections to electronic [71] and muonic energy levels – with both light [79] and heavy [80] nuclei – and to g factors and hyperfine splittings in highly charged ions [73] shows that our model yields an upper estimate of the effect in the HFS of $^3He^+$, and it turned out to be negligible compared to other theoretical contributions.

Analyzing available NP calculation results [79], one can see that the dominant contribution is originated from E1 transitions, fully taken into account in our approach as well. Additionally, we used full relativistic approach for the electronic wave functions. Despite the fact that RPA has been developed for heavy nuclei and should be used with caution for low-Z nuclei, we see a good agreement between our results and those available in the literature, including light nuclei [79]. This, and also the fact that the estimated NP contribution is two orders of magnitude smaller than the total theoretical uncertainty, justify the current use of our model as an approximation. Nonetheless, we conservatively assign the same large 11-kHz uncertainty to NP as to the dominant nuclear effect, namely, the finite-size effect.

With all above mentioned corrections, we obtain a total theoretical value for the hyperfine splitting of $-8\,665\,701(19)$ kHz, in a 2.8 σ tension with the experimental result. We find a difference of 6 parts per million (ppm) for the difference between theoretical and experimental values $\frac{|E_{\rm HFS,exp}-E_{\rm HFS,theo}|}{E_{\rm F}}$, with $E_{\rm F}$ defined in equation (S9).

Relative NP correction	Value
$\Delta E^{\text{NP}}_{\text{HFS}}/E_{\text{HFS}}$ (with F_{BW})	6.51×10^{-9}
$\Delta E^{\text{NP}}_{\text{HFS}}/E_{\text{HFS}}$ (without F_{BW})	1.10×10^{-8}

Table S4: Relative NP corrections to the HFS of $^3\text{He}^+$. The values with and without the smoothing function F_{BW} are given.

2.3 Evaluation of the shielding constant

The combined effect of the nuclear magnetic field and the external magnetic field is expressed by the nuclear shielding contribution. The energy shift due to such diagrams was calculated in Ref. [84]. In the hydrogenlike ion, the shielding contribution is parameterized by replacing the bare nuclear g_I factor by the observable, shielded nuclear g_I factor, $g_I \to g_I(1-\sigma)$, with the shielding constant σ. The factor $(1-\sigma)$ describes the modification of the nuclear magnetic moment through the presence of the bound electron.

Different contributions to the shielding constant σ were calculated following the theoretical methods in Refs. [84, 85]. The leading relativistic shielding term can be given as a closed analytical expression [81]. The second largest correction comes from the nuclear recoil effect, while the dominant QED corrections are the one-loop self-energy diagrams. We found that tabulated results for the Bohr-Weisskopf correction from Ref. [84] for the ions $^{17}\text{O}^{7+}$, $^{43}\text{Ca}^{19+}$ and $^{73}\text{Ge}^{31+}$ can be parametrized as $\alpha(Z\alpha)^4(m_e r_{rms})F_{\text{BW}}(Z\alpha)$ and found that the function $F_{\text{BW}}(Z\alpha)$ only weakly depends on Z. For relatively low Z between $Z=8$ and $Z=32$, $F_{\text{BW}}(Z\alpha)$ varies between -2.0 and -2.4 (using rms radii from [86]). Using values for $F_{\text{BW}}(Z\alpha)$ in this range would correspond to a Bohr-Weisskopf correction for ^3He which is significantly smaller than our estimated uncertainty of the QED correction. We therefore assume the Bohr-Weisskopf correction to be small and give the value obtained in this way in Table S5, with a 100% uncertainty in order to give a conservative upper estimate of the possible error. In a similar way, finite-size corrections, as obtained from tabulated values in Ref. [84], can be parametrized as $\sigma_{\text{FS}} = \alpha(Z\alpha)^4(m_e r_{\text{rms}})F_{\text{FS}}(Z\alpha)$. Similarly to the Bohr-Weisskopf correction, we found that this function $F_{\text{FS}}(Z\alpha)$ varies little between $Z=8$ and $Z=32$, between -2.7 and -3.9. We therefore gave an estimate for the FS correction using above formula for $Z=2$ with typical values observed for $F_{\text{FS}}(Z\alpha)$ which again turned out to be smaller than the uncertainty of the QED effect, and, in particular, much

	$^3\text{He}^+$	Following Refs.
Leading value, all-order $Z\alpha$	0.000 035 521 28	[9–11, 81–83]
FS	-0.000 000 000 006(6)	[24, 84]
1-loop-QED, $\alpha^2(Z\alpha)^3$	0.000 000 000 04(2)	[84]
Recoil $\frac{m}{M}, Z\alpha^2$	-0.000 000 013 93	[9–11, 84]
Bohr-Weisskopf	-0.000 000 000 005(5)	[84]
Total	0.000 035 507 38(3)	

Table S5: Contributions to the shielding constant σ.

smaller than the uncertainty needed to extract the nuclear magnetic moment from the experimental data. Just as in the case of the Bohr-Weisskopf correction, we indicate the estimate for the FS correction obtained in this way in Table S5, with a conservative 100% uncertainty. Attempts to model the numerical data for FS and Bohr-Weisskopf corrections using modified formulas with $\log(Z\alpha)$ terms, $\alpha(Z\alpha)^4(m_e r_{rms})F_{\mathrm{FS/BW}}(Z\alpha)\log(Z\alpha)$, lead to functions $F_{\mathrm{FS/BW}}(Z\alpha)$ which vary much stronger than in the above case. We therefore assume that the leading order terms of these corrections do not contain logarithmic terms. Results are listed in Table S5.

A very recent calculation of the shielding constant [87] is in minor disagreement with our value, primarily due to an updated QED theory of order $\alpha^2(Z\alpha)^3$.

2.4 Determination of the Zemach radius

Instead of comparing experimental and theoretical values for HFS, one can alternatively determine nuclear parameters such as the Zemach radius, assuming the correctness of theory and employing the current experimental value for the nuclear g_I factor. This was performed previously for the Zemach radius of the proton in Ref. [52, 68]. The extraction is based on the HFS formula:

$$E_{\mathrm{HFS,exp}} = E_{\mathrm{HFS,theo}} = E_F\left[A(Z\alpha) + \delta_{\mathrm{FS}} + \delta_{\mathrm{NP}} + \delta_{\mathrm{QED}} + \delta_{\mu\mathrm{VP}} + \delta_{\mathrm{hadVP}} + \delta_{\mathrm{ew}} + \delta_{\mathrm{recoil}}\right] . \tag{S22}$$

Here, E_F is the Fermi hyperfine splitting. This equation can be solved for the nuclear structure-dependent parameters as follows:

$$\delta_{\mathrm{FS}} + \delta_{\mathrm{recoil}} = \frac{E_{\mathrm{HFS,exp}}}{E_F} - A(Z\alpha) - \delta_{\mathrm{NP}} - \delta_{\mathrm{QED}} - \delta_{\mu\mathrm{VP}} - \delta_{\mathrm{hadVP}} - \delta_{\mathrm{ew}} . \tag{S23}$$

The Zemach radius can be adjusted such that above equation is fulfilled. Given a specific set of nuclear model form factors to be used in the leading-order recoil calculation, and assuming the rms charge radius $\sqrt{\langle r^2\rangle_E}$ to be correct, one can determine both r_{Z} and $\sqrt{\langle r^2\rangle_M}$ iteratively, starting from the current literature values. To this end, one employs the definition (S13) of the Zemach radius to determine $\sqrt{\langle r^2\rangle_M}$ from r_{Z}. For the set of model form factors previously used in the proton Zemach radius determination [68], we find a Zemach radius of 2.608(24) fm, with the magnetic radius being 2.15(4) fm. Our extracted Zemach radius disagrees with the value of 2.528(16), determined in Ref. [25] from electron scattering data. The uncertainty accounts for the difference of extracted Zemach radii for all different model form factors used in the leading order recoil calculation, as mentioned above, as well as the uncertainties of all remaining HFS contributions from Table S3.

References

[1] J. Ketter, T. Eronen, M. Höcker, S. Streubel, and K. Blaum, "First-order perturbative calculation of the frequency-shifts caused by static cylindrically-symmetric electric and magnetic imperfections of a Penning trap," Int. J. Mass Spectrom., vol. 358, pp. 1–16, 2014.

[2] S. Rau, High-precision measurement of the deuteron's atomic mass. PhD thesis, Ruperto-Carola University of Heidelberg, Germany, 2020.

[3] L. S. Brown, "Geonium lineshape," Ann. Phys., vol. 159, no. 1, pp. 62–98, 1985.

[4] J. L. Verdu Galiana, Ultrapräzise Messung des elektronischen g-Faktors in wasserstoffähnlichem Sauerstoff. PhD thesis, Johannes Gutenberg-Universität Mainz, Germany, 2004.

[5] J. Zatorski, B. Sikora, S. G. Karshenboim, S. Sturm, F. Köhler-Langes, K. Blaum, C. H. Keitel, and Z. Harman, "Extraction of the electron mass from g-factor measurements on light hydrogenlike ions," Phys. Rev. A, vol. 96, p. 012502, 2017.

[6] C. F. Fischer, G. Gaigalas, P. Jönsson, and J. Bieroń, "GRASP2018 – A Fortran 95 version of the General Relativistic Atomic Structure Package," Comput. Phys. Commun., vol. 237, pp. 184 – 187, 2019.

[7] M. Wang, W. Huang, F. Kondev, G. Audi, and S. Naimi, "The AME2020 atomic mass evaluation (II). Tables, graphs and references," Chin. Phys. C, vol. 45, no. 3, p. 030003, 2021.

[8] E. Tiesinga, P. J. Mohr, D. B. Newell, and B. N. Taylor, "CODATA recommended values of the fundamental physical constants: 2018," Rev. Mod. Phys., vol. 93, p. 025010, Jun 2021.

[9] H. Grotch and R. A. Hegstrom, "Hydrogenic atoms in a magnetic field," Phys. Rev. A, vol. 4, pp. 59–69, Jul 1971.

[10] F. E. Close and H. Osborn, "Relativistic extension of the electromagnetic current for composite systems," Phys. Lett. B, vol. 34, no. 5, pp. 400 – 404, 1971.

[11] R. Faustov, "Magnetic moment of the hydrogen atom," Phys. Lett. B, vol. 33, no. 6, pp. 422 – 424, 1970.

[12] V. M. Shabaev and V. A. Yerokhin, "Recoil correction to the bound-electron g factor in H-like atoms to all orders in αZ," Phys. Rev. Lett., vol. 88, p. 091801, Feb 2002.

[13] M. I. Eides and H. Grotch, "Gyromagnetic Ratios of Bound Particles," Ann. Phys., vol. 260, no. 1, pp. 191 – 200, 1997.

[14] K. Pachucki, "Nuclear mass correction to the magnetic interaction of atomic systems," Phys. Rev. A, vol. 78, p. 012504, Jul 2008.

[15] S. Laporta, "High-precision calculation of the 4-loop contribution to the electron $g-2$ in QED," Phys. Lett. B, vol. 772, no. Supplement C, pp. 232 – 238, 2017.

[16] T. Aoyama, M. Hayakawa, T. Kinoshita, and M. Nio, "Erratum: Tenth-order electron anomalous magnetic moment: Contribution of diagrams without closed lepton loops [Phys. Rev. D 91, 033006 (2015)]," Phys. Rev. D, vol. 96, p. 019901(E), 2017.

[17] A. Czarnecki, M. Dowling, J. Piclum, and R. Szafron, "Two-loop binding corrections to the electron gyromagnetic factor," Phys. Rev. Lett., vol. 120, p. 043203, 2018.

[18] A. Czarnecki, J. Piclum, and R. Szafron, "Logarithmically enhanced Euler-Heisenberg Lagrangian contribution to the electron gyromagnetic factor," Phys. Rev. A, vol. 102, p. 050801, Nov 2020.

[19] V. Debierre, B. Sikora, H. Cakir, N. S. Oreshkina, V. A. Yerokhin, C. H. Keitel, and Z. Harman, "Two-loop virtual light-by-light scattering corrections to the bound-electron g factor," Phys. Rev. A, vol. 103, p. L030802, Mar 2021.

[20] A. Czarnecki, K. Melnikov, and A. Yelkhovsky, "Anomalous magnetic moment of a bound electron," Phys. Rev. A, vol. 63, p. 012509, 2000.

[21] K. Pachucki, A. Czarnecki, U. D. Jentschura, and V. A. Yerokhin, "Complete two-loop correction to the bound-electron g factor," Phys. Rev. A, vol. 72, p. 022108, Aug 2005.

[22] V. A. Yerokhin, C. H. Keitel, and Z. Harman, "Nuclear-size self-energy and vacuum-polarization corrections to the bound-electron g factor," J. Phys. B, vol. 46, no. 24, p. 245002, 2013.

[23] S. G. Karshenboim, "Non-relativistic calculations of the g-factor of a bound electron," Phys. Lett. A, vol. 266, no. 4, pp. 380 – 386, 2000.

[24] S. G. Karshenboim and V. G. Ivanov, "The g factor of the bound muon in medium-Z muonic atoms," Phys. Lett. B, vol. 786, pp. 485–490, 2018.

[25] I. Sick, "Zemach moments of ^{3}He and ^{4}He," Phys. Rev. C, vol. 90, p. 064002, Dec 2014.

[26] N. A. Belov, B. Sikora, R. Weis, V. A. Yerokhin, S. Sturm, K. Blaum, C. H. Keitel, and Z. Harman, "Muonic vacuum polarization correction to the bound-electron g-factor," ArXiv Atomic Physics, 2016. arXiv:1610.01340v1 [physics.atom-ph].

[27] J. Zatorski, N. S. Oreshkina, C. H. Keitel, and Z. Harman, "Nuclear shape effect on the g factor of hydrogenlike ions," Phys. Rev. Lett., vol. 108, p. 063005, Feb 2012.

[28] U. D. Jentschura, A. Czarnecki, K. Pachucki, and V. A. Yerokhin, "Mass measurements and the bound-electron g factor," Int. J. Mass. Spectrom., vol. 251, no. 2-3, pp. 102 – 108, 2006.

[29] V. A. Yerokhin and Z. Harman, "One-loop electron self-energy for the bound-electron g factor," Phys. Rev. A, vol. 95, p. 060501, 2017.

[30] S. Volkov, "Calculating the five-loop qed contribution to the electron anomalous magnetic moment: Graphs without lepton loops," Phys. Rev. D, vol. 100, p. 096004, Nov 2019.

[31] L. Morel, Z. Yao, P. Cladé, and S. Guellati-Khélifa, "Determination of the fine-structure constant with an accuracy of 81 parts per trillion," Nature, vol. 588, pp. 61–65, Dec 2020.

[32] G. Breit, "The magnetic moment of the electron," Nature, vol. 122, pp. 649–649, Oct 1928.

[33] J. Schwinger, "On quantum-electrodynamics and the magnetic moment of the electron," Phys. Rev., vol. 73, pp. 416–417, 1948.

[34] K. Pachucki, U. D. Jentschura, and V. A. Yerokhin, "Nonrelativistic QED approach to the bound-electron g factor," Phys. Rev. Lett., vol. 93, p. 150401, 2004.

[35] S. G. Karshenboim, V. G. Ivanov, and V. M. Shabaev, "Vacuum polarization in a hydrogen-like relativistic atom: g factor of a bound electron," J. Exp. Theor. Phys., vol. 93, p. 477, 2001.

[36] M. E. Peskin and D. V. Schroeder, An Introduction to Quantum Field Theory. Westview Press, 1995.

[37] T. Beier, "The g_j factor of a bound electron and the hyperfine structure splitting in hydrogenlike ions," Phys. Rep., vol. 339, no. 2-3, pp. 79 – 213, 2000.

[38] R. N. Lee, A. I. Milstein, I. S. Terekhov, and S. G. Karshenboim, "Virtual light-by-light scattering and the g factor of a bound electron," Phys. Rev. A, vol. 71, p. 052501, 2005.

[39] A. Petermann, "Fourth order magnetic moment of the electron," Helv. Phys. Acta, vol. 30, pp. 407–408, 1957.

[40] C. M. Sommerfield, "The magnetic moment of the electron," Ann. Phys., vol. 5, no. 1, pp. 26 – 57, 1958.

[41] A. Czarnecki and R. Szafron, "Light-by-light scattering in the lamb shift and the bound electron g factor," Phys. Rev. A, vol. 94, p. 060501(R), Dec 2016.

[42] V. A. Yerokhin and Z. Harman, "Two-loop QED corrections with closed fermion loops for the bound-electron g factor," Phys. Rev. A, vol. 88, p. 042502, Oct 2013.

[43] S. Laporta and E. Remiddi, "The analytical value of the electron $g-2$ at order α^3 in QED," Phys. Lett. B, vol. 379, no. 1, pp. 283 – 291, 1996.

[44] T. Aoyama, M. Hayakawa, T. Kinoshita, and M. Nio, "Revised value of the eighth-order contribution to the electron $g-2$," Phys. Rev. Lett., vol. 99, p. 110406, 2007.

[45] T. Aoyama, M. Hayakawa, T. Kinoshita, and M. Nio, "Tenth-order QED contribution to the electron $g-2$ and an improved value of the fine structure constant," Phys. Rev. Lett., vol. 109, p. 111807, 2012.

[46] D. Nomura and T. Teubner, "Hadronic contributions to the anomalous magnetic moment of the electron and the hyperfine splitting of muonium," Nucl. Phys. B, vol. 867, no. 2, p. 236, 2013.

[47] A. Kurz, T. Liu, P. Marquard, and M. Steinhauser, "Hadronic contribution to the muon anomalous magnetic moment to next-to-next-to-leading order," Phys. Lett. B, vol. 734, p. 144, 2014.

[48] J. Prades, E. de Rafael, and A. Vainshtein, vol. 20 of Advanced Series on Directions in High Energy Physics, ch. 9, pp. 303–317. Singapore: World Scientific, 2010.

[49] V. M. Shabaev, "Hyperfine structure of hydrogen-like ions," J. Phys. B, vol. 27, pp. 5825–5832, dec 1994.

[50] N. Stone, "Table of nuclear magnetic dipole and electric quadrupole moments," At. Data Nucl. Data Tables, vol. 90, no. 1, pp. 75 – 176, 2005.

[51] A. V. Volotka, V. M. Shabaev, G. Plunien, and G. Soff, "Nuclear size correction to the hyperfine splitting in low-Z hydrogen-like atoms," Eur. Phys. J. D, vol. 23, no. 1, pp. 51–56, 2003.

[52] A. V. Volotka, High-precision QED calculations of the hyperfine structure in hydrogen and transition rates in multicharged ions. PhD thesis, Technische Universität Dresden, 2006.

[53] A. C. Zemach, "Proton structure and the hyperfine shift in hydrogen," Phys. Rev., vol. 104, pp. 1771–1781, Dec 1956.

[54] K. Pachucki, V. A. Yerokhin, and P. Cancio Pastor, "Quantum electrodynamic calculation of the hyperfine structure of ^{3}He," Phys. Rev. A, vol. 85, p. 042517, Apr 2012.

[55] S. G. Karshenboim, "Nuclear structure-dependent radiative corrections to the hydrogen hyperfine splitting," Phys. Lett. A, vol. 225, no. 1, pp. 97 – 106, 1997.

[56] J. S. McCarthy, I. Sick, and R. R. Whitney, "Electromagnetic structure of the helium isotopes," Phys. Rev. C, vol. 15, pp. 1396–1414, Apr 1977.

[57] H. De Vries, C. De Jager, and C. De Vries, "Nuclear charge-density-distribution parameters from elastic electron scattering," At. Data Nucl. Data Tables, vol. 36, no. 3, pp. 495–536, 1987.

[58] T. Kinoshita, "NRQED approach to the hyperfine structure of the muonium ground state," ArXiv High Energy Physics - Phenomenology (hep-ph), 1998. arXiv:hep-ph/9808351v1.

[59] V. A. Yerokhin and U. D. Jentschura, "Self-energy correction to the hyperfine splitting and the electron g factor in hydrogenlike ions," Phys. Rev. A, vol. 81, p. 012502, 2010.

[60] S. G. Karshenboim and V. G. Ivanov, "Hyperfine structure of the ground and first excited states in light hydrogen-like atoms and high-precision tests of QED," Eur. Phys. J. D, vol. 19, pp. 13–23, Apr 2002.

[61] S. G. Karshenboim, V. G. Ivanov, and V. M. Shabaev, "Polarization of vacuum in a hydrogen-like relativistic atom: Hyperfine structure," J. Exp. Theor. Phys., vol. 90, pp. 59–65, Jan 2000.

[62] S. G. Karshenboim, "Leading logarithmic corrections and uncertainty of muonium hyperfine splitting calculations," Z. Phys. D, vol. 36, pp. 11–15, Mar 1996.

[63] M. I. Eides, "Weak-interaction contributions to hyperfine splitting and lamb shift in light muonic atoms," Phys. Rev. A, vol. 85, p. 034503, Mar 2012.

[64] G. T. Bodwin, D. R. Yennie, and M. A. Gregorio, "Recoil effects in the hyperfine structure of QED bound states," Rev. Mod. Phys., vol. 57, pp. 723–782, Jul 1985.

[65] G. T. Bodwin and D. R. Yennie, "Some recoil corrections to the hydrogen hyperfine splitting," Phys. Rev. D, vol. 37, pp. 498–523, Jan 1988.

[66] C. E. Carlson, Proton Structure Corrections to Hydrogen Hyperfine Splitting, pp. 93–107. Springer Berlin Heidelberg, 2008.

[67] C. E. Carlson, V. Nazaryan, and K. Griffioen, "Proton-structure corrections to hyperfine splitting in muonic hydrogen," Phys. Rev. A, vol. 83, p. 042509, Apr 2011.

[68] A. V. Volotka, V. M. Shabaev, G. Plunien, and G. Soff, "Zemach and magnetic radius of the proton from the hyperfine splitting in hydrogen," Eur. Phys. J. D, vol. 33, pp. 23–27, Apr 2005.

[69] G. Plunien, B. Müller, W. Greiner, and G. Soff, "Nuclear polarization contribution to the Lamb shift in heavy atoms," Phys. Rev. A, vol. 39, pp. 5428–5431, 1989.

[70] L. Labzowsky and A. Nefiodov, "Analytic evaluation of the nuclear polarization contribution to the energy shift in heavy ions," Phys. Lett. A, vol. 188, no. 4, pp. 371 – 375, 1994.

[71] A. Nefiodov, L. Labzowsky, G. Plunien, and G. Soff, "Nuclear polarization effects in spectra of multicharged ions," Phys. Lett. A, vol. 222, no. 4, pp. 227–232, 1996.

[72] A. Nefiodov, G. Plunien, and G. Soff, "Nuclear-polarization effect to the hyperfine structure in heavy multicharged ions," Phys. Lett. B, vol. 552, no. 1, pp. 35–40, 2003.

[73] A. V. Volotka and G. Plunien, "Nuclear polarization study: New frontiers for tests of QED in heavy highly charged ions," Phys. Rev. Lett., vol. 113, p. 023002, 2014.

[74] H. Cakir, N. S. Oreshkina, I. A. Valuev, V. Debierre, V. A. Yerokhin, C. H. Keitel, and Z. Harman, "Improved access to the fine-structure constant with the simplest atomic systems," ArXiv Atomic Physics, 2020. arXiv:2006.14261v1 [physics.atom-ph].

[75] G. Colò, L. Cao, N. Van Giai, and L. Capelli, "Self-consistent RPA calculations with Skyrme-type interactions: The skyrme_rpa program," Comput. Phys. Commun., vol. 184, no. 1, pp. 142–161, 2013.

[76] I. A. Valuev, Z. Harman, C. H. Keitel, and N. S. Oreshkina, "Skyrme-type nuclear interaction as a tool for calculating the finite-nuclear-size correction to atomic energy levels and the bound-electron g factor," Phys. Rev. A, vol. 101, p. 062502, Jun 2020.

[77] I. A. Valuev, G. Colò, X. Roca-Maza, C. H. Keitel, and N. S. Oreshkina, "Evidence against nuclear polarization as source of fine-structure anomalies in muonic atoms," 2022. arXiv:2201.09638 [physics.atom-ph].

[78] N. Michel, N. S. Oreshkina, and C. H. Keitel, "Theoretical prediction of the fine and hyperfine structure of heavy muonic atoms," Phys. Rev. A, vol. 96, p. 032510, 2017.

[79] C. Ji, S. Bacca, N. Barnea, O. J. Hernandez, and N. N. Dinur, "Ab initio calculation of nuclear-structure corrections in muonic atoms," J. Phys. G, vol. 45, p. 093002, aug 2018.

[80] P. Bergem, G. Piller, A. Rueetschi, L. A. Schaller, L. Schellenberg, and H. Schneuwly, "Nuclear polarization and charge moments of ^{208}Pb from muonic x rays," Phys. Rev. C, vol. 37, pp. 2821–2833, Jun 1988.

[81] E. A. Moore, "Relativistic chemical shielding: formally exact solutions for one-electron atoms of maximum total angular momentum for any principal quantum number," Mol. Phys., vol. 97, no. 3, pp. 375–380, 1999.

[82] N. C. Pyper, "Relativistic theory of nuclear shielding in one-electron atoms 1. theoretical foundations and first-order terms," Mol. Phys., vol. 97, no. 3, pp. 381–390, 1999.

[83] N. C. Pyper and Z. C. Zhang, "Relativistic theory of nuclear shielding in one-electron atoms 2. analytical and numerical results," Mol. Phys., vol. 97, no. 3, pp. 391–413, 1999.

[84] V. A. Yerokhin, K. Pachucki, Z. Harman, and C. H. Keitel, "QED calculation of the nuclear magnetic shielding for hydrogenlike ions," Phys. Rev. A, vol. 85, p. 022512, Feb 2012.

[85] A. Rudziński, M. Puchalski, and K. Pachucki, "Relativistic, QED, and nuclear mass effects in the magnetic shielding of ^{3}He," J. Chem. Phys., vol. 130, no. 24, p. 244102, 2009.

[86] I. Angeli and K. Marinova, "Table of experimental nuclear ground state charge radii: An update," At. Data Nucl. Data Tables, vol. 99, no. 1, pp. 69 – 95, 2013.

[87] D. Wehrli, A. Spyszkiewicz-Kaczmarek, M. Puchalski, and K. Pachucki, "QED Effect on the Nuclear Magnetic Shielding of ^{3}He," Phys. Rev. Lett., vol. 127, p. 263001, Dec 2021.
